# von Willebrand Factor is elevated in HIV patients with a history of thrombosis

**DOI:** 10.3389/fmicb.2015.00180

**Published:** 2015-03-11

**Authors:** Lennert W. J. van den Dries, Rob A. Gruters, Sascha B. C. Hövels–van der Borden, Marieke J. H. A. Kruip, Moniek P. M. de Maat, Eric C. M. van Gorp, Marchina E. van der Ende

**Affiliations:** ^1^Department of Viroscience, Erasmus MC, University Medical CenterRotterdam, Netherlands; ^2^Department of Neurology, Maasstad HospitalRotterdam, Netherlands; ^3^Division of Hematology, Department of Internal Medicine, Erasmus MC, University Medical CenterRotterdam, Netherlands; ^4^Division of Infectious Diseases, Department of Internal Medicine, Erasmus MC, University Medical CenterRotterdam, Netherlands

**Keywords:** LPS, von Willebrand Factor, thrombosis, HIV, coagulation, immune activation

## Abstract

**Background:** Arterial and venous thrombotic events are more prevalent in HIV infected individuals compared to the general population, even in the era of combination antiretroviral therapy. Although the mechanism is not fully understood, recent evidence suggests a role for chronic immune activation.

**Methods:** We reviewed the Dutch National HIV registry database for HIV infected patients in Rotterdam with a history of arterial or venous thrombosis and calculated the incidence. We collected samples from patients with and without thrombosis and compared plasma levels of lipopolysaccharide (LPS), LPS binding protein (LBP), soluble CD14 (sCD14), and von Willebrand Factor antigen level (vWF).

**Results:** During a 10-year period, a total of 60 documented events in 14,026 person years of observation (PYO) occurred, resulting in an incidence rate of 2.50, 2.21, and 4.28 for arterial, venous and combined thrombotic events per 1000 PYO, respectively. The vWF was elevated in the majority of study subjects (mean 2.36 SD ± 0.88 IU/ml); we found a significant difference when comparing venous cases to controls (mean 2.68 SD ± 0.82 IU/ml vs. 2.20 SD ± 0.77 IU/ml; *p* = 0.024). This difference remained significant for recurrent events (mean 2.78 SD ± 0.75; *p* = 0.043). sCD14 was positively correlated with LPS (*r* = 0.255; *p* = 0.003).

**Conclusion:** The incidence of venous thrombosis was two-fold higher in HIV infected patients compared to age-adjusted data from general population cohort studies. We couldn't find a clear association between immune activation markers to either arterial or venous thrombotic events. We observed a marked increase in vWF levels as well as a correlation of vWF to first and recurrent venous thrombo-embolic events. These findings suggest that HIV infection is an independent risk factor for coagulation abnormalities and could contribute to the observed high incidence in venous thrombosis. This could be a reason to prolong anti-thrombotic treatment in HIV patients with a history of thrombosis.

## Introduction

The delicate interaction between inflammation and coagulation has long been recognized and persists to play a pivotal role in numerous infections such as sepsis and viral hemorrhagic diseases. In chronic HIV infection, the hemostatic balance is tipped toward a more pro-coagulant status, resulting in thrombosis (Goeijenbier et al., 2012). This is supported by both epidemiological and experimental data.

The annual risk on venous thrombosis in a representable European population below the age of 60 is slightly over 1 per 1000 person years of observation (PYO) (Oger, 2000; Naess et al., 2007). For arterial thrombosis, the annual risk is higher with incidence rates between 2 and 3 per 1000 PYO for myocardial infarction and around 0.65 per 1000 PYO for stroke (Vaartjes et al., 2013; Van Oeffelen et al., 2014). It is likely that HIV infection independently adds to this risk (Shen and Frenkel, 2004). A nationwide study comprising of 4333 HIV infected individuals reported an incidence of venous thrombosis in 3.2 per 1000 PYO (Rasmussen et al., 2011). In addition, events occur at a significantly younger age compared to the control population (Copur et al., 2002; Malek et al., 2011). Similar trends have been observed in myocardial infarction (Esser et al., 2013), stroke (Marcus et al., 2014) and peripheral atherosclerosis (Periard et al., 2008). A large tri-continental study determined an incidence of 5.7 per 1000 PYO for first cardio- or cerebrovascular event in a relatively young cohort (d'Arminio et al., 2004) with cumulative exposure to cART as a major contributor (Friis-Møller et al., 2003a,b).

The exact pathophysiology of increased thrombotic activity in HIV remains unknown but recent publications advocate a role for chronic immune activation. In this hypothesis, HIV infection causes a loss of mucosal integrity in the gut together with CD4 T-cell depletion in local lymphoid tissue. This results in translocation of microbial products from the lumen to the circulation (Brenchley et al., 2006). Bacterial endotoxins such as LPS are subsequently bound to pattern recognition receptors and trigger a potent inflammatory response in monocytes and macrophages (Kristoff et al., 2014; Shan and Siliciano, 2014). LPS, LBP and soluble CD14 levels have been found to correlate with a hypercoagulable state in chronic HIV, with or without combined cART (Jong et al., 2010; Sandler et al., 2011; Romero-Sánchez et al., 2012; Funderburg, 2014). Although it is conceivable that immune activation accelerates clot formation, the exact mechanism remains to be elucidated.

In this study we addressed HIV as a common risk factor for both arterial and venous thrombosis and investigated chronic immune activation as the proposed driving mechanism. To test this hypothesis, we assessed the incidence of venous and arterial thrombotic events in a chronically infected HIV population. We compared coagulation (vWF), microbial translocation (LPS and LBP) and inflammatory parameters (sCD14) of patients with a past thrombotic event to patients without an event. We hypothesized that HIV infected individuals with a past thrombotic event have a higher exposure to microbial-driven immune activation.

## Materials and methods

### Patients and study design

On February 25th 2013, we retrieved information from the Stichting HIV Monitoring (SHM) database, described elsewhere, which includes anonymized data obtained from treated and untreated HIV-infected patients, who have been followed in or after 1996 in our hospital (Van Sighem et al., 2013). Cases were defined as patients with a thrombotic event and a presumed or definite preceding HIV diagnosis. A preceding HIV status was presumed when the CD4 cell count was <200/mm^3^ within 1 year of the thrombotic event. Venous thrombo-embolic events included deep venous thrombosis (DVT), diagnosed by compression ultrasonography; or pulmonary embolism (PE), diagnosed by Computer Tomography (CT) pulmonary angiography. Arterial thrombo-embolic events included myocardial infarction (MI), diagnosed by electrocardiogram and cardiac biomarkers; cerebrovascular incident (CVA) or ischemic attack (TIA), diagnosed by neurological examination in combination with CT scan results; and claudication intermittens (CI) diagnosed by the ankle-brachial index. Cases were compared to randomly selected controls, i.e., patients with HIV infection but no thrombo-embolic event in their history. This control population was comparable to the general HIV population in Rotterdam with respect to age and sex. All cases and controls were offered a questionnaire concerning classical risk factors. This research was approved by the local ehtics committee, patients had to sign an informed consent document to participate.

### LPS, LBP, sCD14, and vWF measurements

We collected plasma from 65 cases and 65 control patients for analysis of LPS, LBP, sCD14, and vWF. The patient blood samples were collected in Ethylene diamine tetra acetic acid (EDTA). These were initially intended for viral load analysis and stored at −80°C. The mean time from thrombotic event to blood sample collection was 6.2 years (SD ± 4.7). Samples were thawed only once to prevent protein degradation. LPS levels were measured using the Pyros Kinetix Flex® chromogenic endotoxin detection system (Associates of Cape Cod). Plasma samples were heat inactivated at 60°C and subsequently diluted with LPS free LAL H_2_O at concentrations varying from 1:20 to up to 1:400. Exact LPS quantities were derived from a standard curve of known control endotoxin concentrations. LBP was measured using a LBP ELISA kit® (Hycult biotech); soluble CD14 was measured using the Human sCD14 Quantikine ELISA Kit® (R&D systems); vWF antigen levels were measured with an in-house ELISA using DAKOPATTS antibodies and compared to the vWF antigen levels to commercially available pooled plasma from 20 or more otherwise healthy donors (CRYOcheck™).

### Data analysis

For the calculation of incidence, we used available SHM data on all HIV patients in care at the Erasmus MC from February 25th 2003 to February 25th 2013. The incidence rate of an event was calculated as the number of documented cases occurring in a 10 year time period, divided by the total amount of unique person-years contributed to the cohort. Data was censored when an event occurred, when the patient died or on the index date, whichever came first. Cases with both arterial and venous events were censored on whichever date came first, but were included in both calculations.

Normality of data was assessed by a Shapiro Wilk test and inspection of Q-Q Plots. Homogeneity of variance was assessed by Levene's Test for Equality of Variances. An independent *T*-test or Man-Whitney was performed for continuous variables when appropriate. vWF was normally distributed, and sCD14, LBP, and LPS showed a non-parametric distribution. For categorical variables, a Chi-Square test was applied. Correlation analysis was performed using the Spearman's Rank-order test. A significance level of 0.05 was applied. All statistical analysis was performed using SPSS software, version 21 (IBM corp ©). Graphs were constructed using GraphPad Prism software, version 6.

## Results

From February 25th 2003 to February 25th 2013, a total of 2731 unique patients were registered in the Rotterdam SHM database, representing 14,026 person years. A total of 60 thrombotic events occurred during this 10-year period. The overall incidence of both arterial and venous thrombotic events combined was 4.28 per 1000 PYO. These rates were 2.50 and 2.21 for arterial events and venous thrombotic events, respectively. The incidence of patients with a recurrent event was 0.93 in the arterial group and 0.57 in the venous group per 1000 PYO when divided by the total amount of person years. When divided by the corresponding amount of person years in the specific event group only, the incidence increased to 95 in the arterial group and 73 in the venous group per 1000 PYO. In the group that had experienced a venous thrombosis, the incidence of patients that had a recurrence of a venous event was 30% after a period of 3 years.

**Figure 1:**
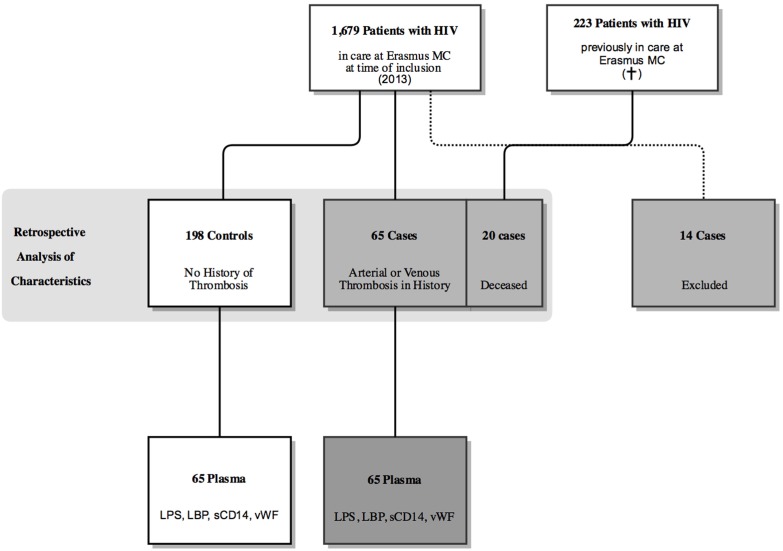
**Flow chart depicting the selection of patients for this study**.

At the time of inclusion on February 25th 2013, a total of 1679 HIV-infected patients were still in care out of which 79 were reported with a thrombotic event (see Figure 1). Of these 79 patients, 10 patients were excluded based on a negative HIV test at the time of event and 4 patients refused participation, resulting in 65 available cases. In addition, we searched the database for patients that died between April 1st 2002 and November 22nd 2012 and had a history of thrombosis. In total, 223 patients died during this period of which 20 had a documented thrombotic event in the past. Of these 85 patients, 37 were diagnosed with a PE or DVT; 41 with a MI, CVA or CI; and 7 had endured both a venous and arterial event.

A random selection of the patients registered in the Rotterdam SHM database (as described earlier) was applied to select a control group of 198 HIV-infected patients. This group was comparable to the whole HIV population at the Erasmus Medical Center in respect to age and sex. When compared to the 198 controls at the time of observation, cases with arterial and venous events were significantly older [46.85 SD ± 11.10 vs. 57.83 SD ± 11.01 years *p* < 0.001 (arterial) vs. 52.80 SD ± 11.21 *p* = 0.007 (venous)], more likely to be of Caucasian descent [46.5% vs. 70.7% *p* = 0.005 (arterial) vs. 67.6% *p* = 0.018 (venous)] and were more likely to have history of hypertension [8.1% vs. 39% *p* < 0.001 (arterial) vs. 21.6% *p* = 0.013 (venous)] and malignancy [6.1% vs. 19.5% *p* = 0.005 (arterial) vs. 21.6% *p* = 0.002 (venous)]. Compared to controls, an arterial event was specifically associated with male sex (71.2% vs. 90.2% *p* = 0.011), a positive family history for cardiovascular events (17.7% vs. 39.0% *p* = 0.002) and diabetes mellitus (4.5% vs. 22.0% *p* < 0.001). There was no difference between cases and controls for traditional risk factors such as a high body mass index, smoking or immobilization. No difference was found for HIV related risk factors such as a low CD4 nadir count or protease inhibitor (PI) use (Table 1).

**Table 1:** **Patient characteristics**.

	**Controls (*N* = 198)**		**Cases (85)**		***P*-value**
**Males (%)**	141	(71.2)	70	(82.4)	0.049
Venous events only			28	(75.7)	0.579
Arterial events only			37	(90.2)	0.011
Both events only			5	(71.4)	0.990
**Age (SD)^a^**	47	(11.1)	56	(11.0)	<0.001
Venous events only			53	(11.2)	0.007
Arterial events only			58	(11.0)	<0.001
Both events only			58	(7.7)	0.008
**Caucasian (%)**	92	(46.5)	61	(71.8)	<0.001
Venous events only			25	(67.6)	0.018
Arterial events only			29	(70.7)	0.005
Both events only			5	(71.4)	0.005
**CD4 nadir (IQR)**	180	(67.5–300)	170	(65–275)	0.852
Venous events only			190	(70–275)	0.931
Arterial events only			170	(60–315)	0.874
Both events only			140	(40–260)	0.575
**HIV RNA undetectable (%)^a^**	168	(84.8)	78	(91.8)	0.114
Venous events only			35	(94.6)	0.113
Arterial events only			36	(87.8)	0.626
Both events only			7	(100)	0.265
**cART use^a^**	183	(92.4)	80	(94.1)	0.610
Venous events only			35	(94.6)	0.640
Arterial events only			38	(92.7)	0.954
Both events only			7	(100)	0.449
**PI use^a^**	58	(29.3)	31	(36.5)	0.233
Venous events only			13	(35.1)	0.477
Arterial events only			16	(39.0)	0.220
Both events only			2	(28.6)	0.967

a *At time of inclusion or at death; SD, Standard Deviation; IQR, Inter Quartile Range; cART, Combination Antiretroviral Therapy; PI, Protease Inhibitor*.

**Figure 2:**
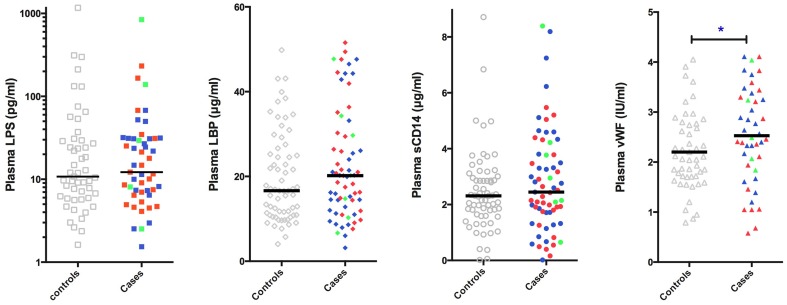
**Plasma levels of LPS (*N* = 101), LBP (*N* = 130), sCD14 (*N* = 130), and vWF antigen (*N* = 93) in chronic HIV infected individuals with or without a history of arterial or venous thrombotic disease**. Horizontal bar represents median for LPS, LBP, and sCD14; and mean in vWF. Cases had a significantly higher vWF antigen level as compared to controls. Gray, controls without a thrombotic event; red, arterial events; blue, venous events; green, both arterial and venous events. LPS, Lipopolysaccharide; LBP, LPS binding protein; sCD14, soluble CD14; vWF, von Willebrand Factor antigen level. ^*^p = 0.024 for venous events vs. controls.

**Figure 3:**
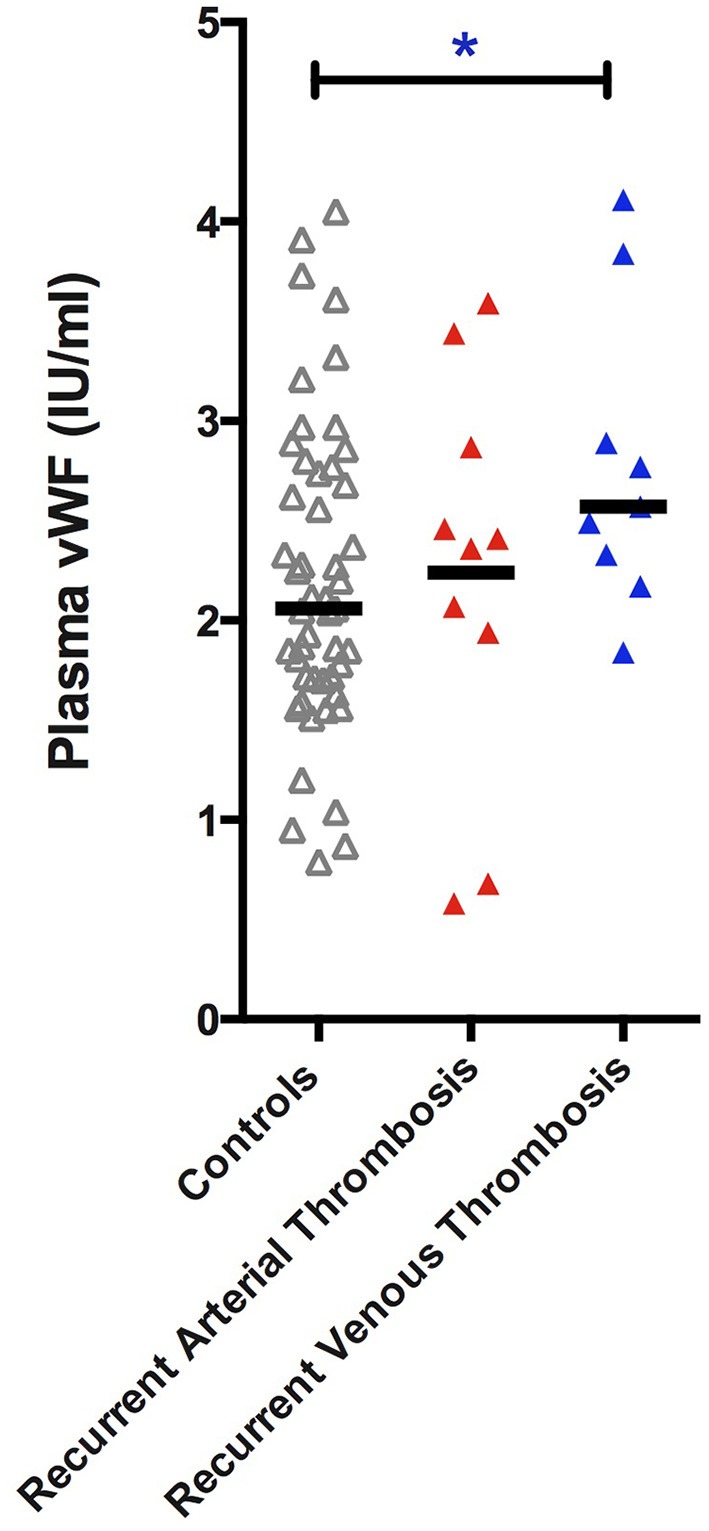
**vWF antigen in individuals with HIV without thrombosis compared to HIV infected individuals with a recurrent venous or arterial trombotic event**. Patients with a recurrent venous thrombotic event had a statistically significant higher vWF (*p* = 0.043). vWF, von Willebrand Factor antigen level.

**Figure 4:**
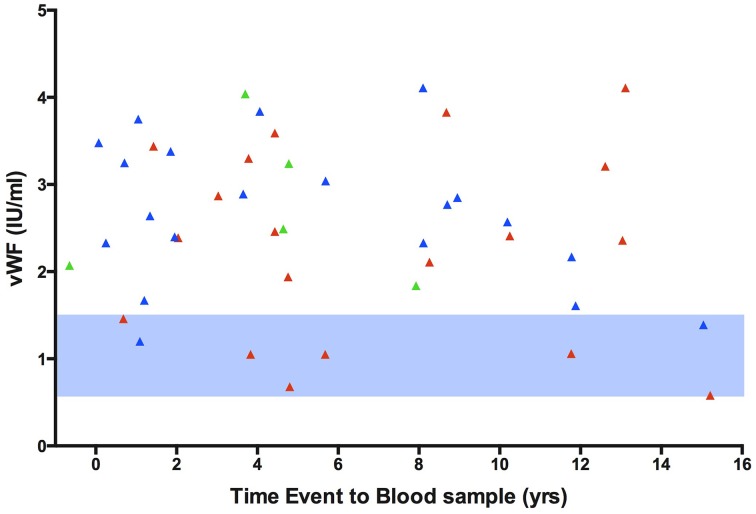
**Time from event to blood sample collection plotted against vWF antigen level in HIV infected individuals with an arterial or venous event**. The level of vWF antigen is considerably higher than the reference value. vWF, von Willebrand Factor antigen. Red, arterial events; blue, venous events; green, both arterial and venous events. Light blue area, reference value for vWF antigen level.

For patient characteristics of subjects that underwent analysis of sCD14, LPS, LBP, and vWF please see Supplementary Table 1. Patients with a thrombotic event in the past had a higher mean vWF antigen level when compared to controls (venous: 2.68 SD ± 0.82 IU/ml; arterial: 2.31 SD ± 1.10 IU/ml; both: 2.74 SD ± 0.90 IU/ml; controls: 2.20 SD ± 0.77 IU/ml). The difference in vWF antigen levels was significant in patients with a past venous event compared to controls (*p* = 0.024, Independent samples *T*-test) (Figure 2), which remained significant when comparing only recurrent events to controls (2.78 SD ± 0.75; *p* = 0.043 Independent Samples *T*-test, Figure 3). The significance of difference in vWF antigen level between cases combined vs. controls varied upon the applied statistical test (*p* = 0.047, Mann Whitney; *p* = 0.071 Independent samples *T*-test). There was no clear connection between time since thrombotic event and the level of vWF antigen (see Figure 4). There was no significant difference between cases and controls in sCD14 (median: 2.45 IQR ± 2.08 vs. 2.31 IQR ± 1.36; *p* = 0.341) and LBP (median: 20.19 IQR ± 17.56 vs. 16.63 IQR ± 15.05 μ/ml; *p* = 0.264) as well as LPS (median: 12.19 IQR ± 24.70 vs. 10.76 IQR ± 23.58) although values tended to be higher in cases (Figure 2).

**Figure 5:**
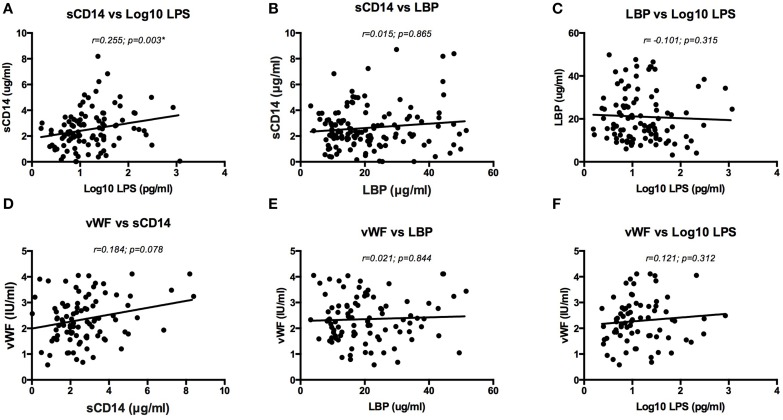
**Correlation analysis of sCD14 vs LPS (log-transformed) **(A)**, sCD14 vs LBP **(B)**, LBP vs LPS (log-transformed) **(C)**, vWF vs sCD14 **(D)**, vWF vs LBP **(E)** and vWF vs LPS (log-transformed) **(F)****. A Spearman's Rank-order test was used for analysis. Only sCD14 and LPS (log-transformed) had a statistically significant correlation **(A)**. There was a weak trend toward positive correlation between vWF and sCD14 **(D)**. LPS, Lipopolysaccharide; LBP, LPS binding protein; sCD14, soluble CD14; vWF, von Willebrand Factor antigen level.

The only association detected was a positive correlation between levels of soluble CD14 and log 10 levels of LPS (*p* = 0.003; *r* = 0.255, Figure 5A). There was also a weak trend toward positive correlation between plasma levels of vWF and sCD14 (*p* = 0.078; *r* = 0.184, Figure 5D). The four other combinations of markers (vWF vs. LBP; vWF vs. log 10 LPS; sCD14 vs. LBP; LBP vs. log 10 LPS) were not correlated (all *p* > 0.1) (Figures 5B,C,E,F).

## Discussion

Our results confirm previous observations of thrombosis being a prevalent co-morbidity in the HIV population, even in a cohort with prolonged c-ART treatment. Comparable to uninfected individuals, traditional risk factors such as diabetes, malignancy, hypertension and a positive family history continue to contribute to the development of an event, but additional factors play a role. This study was aimed to investigate the incidence of arterial and venous thrombosis in patients living with chronic HIV infection. In addition we wanted to address what factors predispose HIV patients for the occurrence of a thrombotic event and by which mechanism.

To answer the first question, we performed a retrospective analysis. The incidence of arterial and thrombotic events combined was 4.28 per 1000 PYO. We found an incidence of 2.21 per 1000 PYO of venous thrombotic events, which is more than two-fold higher compared to healthy controls from large cohort studies (Naess et al., 2007). This result corroborates with literature where the risk on venous thrombotic disease is reported to be 2–10-fold increased (Klein et al., 2005; Crum-Cianflone et al., 2008). The variation in observed incidences between studies is probably related to the nature of these studies, mostly retrospective, the difference in diagnostic criteria and cohort characteristics such as immune status of patients. An increasing amount of evidence in the literature supports the notion that HIV status is associated with increased risk on myocardial infarction, stroke and venous thrombo-embolism (Micieli et al., 2007). With the aging of HIV cohorts and related increased exposure to cART, it remains important to re-evaluate this incidence rate. The incidence rate for arterial events in our study was lower than previously found. Compared to the Data Collection on Adverse Events of Anti-HIV Drugs (DAD) Study Group, the incidence in our study is two-fold lower (d'Arminio et al., 2004). The prevalence of risk factors such as smoking, diabetes and hypertension, were comparable in both studies. One difference is a high use of PI's in the DAD study cohort. PI's are known to cause metabolic abnormalities and could add in disease severity, although a direct causal relationship with arterial disease remains controversial (Sklar and Masur, 2003).

In HIV infection, several well-defined coagulation abnormalities exist, such as Activated Protein C resistance, Protein S deficiency, increased D-dimer, Tissue Factor expression on monocytes and increased levels of vWF. In this study, we choose to examine vWF as a prognostic marker to investigate the predisposition to a thrombotic event. This protein is crucial in both primary and secondary hemostasis. It is produced almost exclusively in the endothelium and is stored as large multimers in vesicles called Weibel Palade bodies. The vWF antigen level in HIV patients was two-fold higher compared to the reference material (i.e., healthy donors), which implies an increased coagulation potential in all HIV infected patients. Several mechanisms could potentially result in a high level of vWF antigen. A delayed clearance of vWF, as is seen in conditions such as thrombotic thrombocytopenic purpura (TTP) and hemolytic uremic syndrome (HUS), is unlikely the cause of high vWF antigen in HIV. The activity of ADAMTS13, the metalloproteinase responsible for clearing vWF, can be lower in HIV, but does not reach the levels needed for overt pathology (Jong et al., unpublished data; Badenhorst et al., 2009). In addition, antibodies against ADAMTS13 are incidental in HIV related TTP (Gunther et al., 2007). It seems more logical to attribute the high vWF to increased transcriptional activity in endothelial cells due to chronic immune activation (Baker, 2013). The production, storage and exocytosis of vWF are regulated through intrinsic and extrinsic factors such as, but not limited to nitric oxide, hypoxia, histamine, thrombin and other secretagogues. LPS, for instance, could be a potent stimulator of endothelial cells. Therefore, we explored the possibility of immune activation as a driving factor behind arterial and venous thrombosis. It is likely that monocytes rather than T-cells are responsible for the initial immunologic response to LPS translocation, considering these cells express CD14 and TLR4, the receptors for LPS. Although markers of coagulation and immune activation are partially restored upon initiation of cART, a complete normalization fails to appear (Jong et al., 2009; Funderburg, 2014). In the SMART study, a study aimed to investigate the benefit of a CD4 guided approach on treatment (El-Sadr et al., 2006), markers of inflammation and coagulation such as IL-6, D-dimer and sCD14 were shown to be excellent predictors of atherosclerosis and mortality (Kuller et al., 2008; Sandler et al., 2011; Kelesidis et al., 2012). If immune activation is chronic and predisposes patients for morbidity, we expected to find higher baseline levels of these markers in patients with a thrombotic event in the past. Although levels of immune activation tended to be at the high end in all patients, we could not detect a significant difference between cases and controls. Correlation analysis revealed only a significant correlation between sCD14 and LPS (Figure 5A). This could represent the increased inflammatory response of monocytes on microbial products. It is reasonable to assume that immunologic and hemostatic factors can mutually influence each other. However, all the other associations we describe in this manuscript were non-significant.

Taken together, these data support the hypothesis of an increased activity in coagulation, although the exact mechanism remains to be elucidated. The presence of endothelial cell activation, increased fibrin formation and decreased anticoagulation, as observed in HIV infected patients, is compatible with a pro-thrombotic state (Jong et al., 2010; Arildsen et al., 2013) and mirror markers of immune activation (Eastburn et al., 2011). Additive pro-coagulant mechanisms such as increased tissue factor activity on monocytes as proposed by Funderburg et al. (2010) could be involved alongside endothelial cell activation. In the end, the model of chronic immune activation as proposed by Brenchley et al. (2006), has given rise to new insights in the pro-thrombotic state in HIV. In the meanwhile, we should consider HIV as an independent risk factor for thrombosis. Possible strategies to further investigate immune activation as a causative mechanism could include the use of anti-inflammatory agents or early initiation of therapy. Further research on the specific interaction between endothelial cells and monocytes during HIV infection would be especially interesting. Caution has to be made in conferring primary prevention of thrombosis in HIV patients, since anti-coagulation has potentially serious side effects. It may be worth considering extending secondary prophylaxis in HIV patients with a history of venous thrombosis, since the recurrence rate is relatively high. Although we found a significant difference in vWF between venous recurrences and controls, the wide distribution of values prevents an accurate discrimination between these two, limiting vWF as a predictor for venous thrombosis.

The strength of our study is the detailed baseline demographic and clinical data recorded from a well-characterized population in the SHM cohort. Our study does have several limitations. The study is retrospective, relying largely on the quality of existing data. We are aware of the fact that our data reflect only documented thrombotic events, so the incidence calculated in our study probably underestimates the real number. The ideal study would be a prospective study with comparable person years of inclusion and blood samples before and after an event. We realize this is a laborious task considering practical issues. In addition, HIV-positive patients were mostly on cART, so the individual contribution of HIV infection and cART to immune activation biomarkers could not be distinguished. Our experiments could not establish a relationship between LPS, LBP, sCD14 and event status. A possible explanation could be the large interval between occurrence of the event and measurement of the parameters (mean = 6.2 years later). We would expect a higher level of immune activation markers around the time of the event. Although microbial translocation has gained much attention as a potential driver for immune activation, it should be stated other mechanisms have not been addressed in this study. We cannot exclude other potential mechanisms such as residual viral replication, CMV seropositivity and pyroptosis (Doitsh et al., 2014) as a driver for coagulation abnormalities.

In conclusion, our study confirms previous findings that HIV-infection results in a pro-thrombotic state, reflected by a high incidence in venous thrombotic events and a high percentage that experiences a recurrence. Although we did not see a clear association between markers of immune activation to event status, we did encounter a significant difference in vWF levels between patients with a past venous thrombo-embolism and those without. These data support the rationale to extend anticoagulant therapy once venous thrombosis has occurred. However, further investigation on, e.g., fVIII and other pro-coagulants are needed. The wide distribution of vWF in our patient groups does not support the use of this marker as a clinical predictor for recurrent thrombosis in HIV patients.

### Conflict of interest statement

The authors declare that the research was conducted in the absence of any commercial or financial relationships that could be construed as a potential conflict of interest.
