# Supplementary material for: von Willebrand Factor is elevated in HIV patients with a history of thrombosis
Source: Front Microbiol. 2015 Mar 11;6:180. doi: 10.3389/fmicb.2015.00180 (PMC4356086; doi:10.3389/fmicb.2015.00180)
Supplement: Supplementary file 1 [file DataSheet1.DOCX]

**Supplementary table 1.** Patient characteristics of the 130 patients of whom samples were obtained. Cases and controls were age- and sex matched as a group.
